# Experience of Nurses in Hemodialysis Care: A Phenomenological Study

**DOI:** 10.3390/jcm7020030

**Published:** 2018-02-11

**Authors:** Hosien Shahdadi, Mozhgan Rahnama

**Affiliations:** Department of Nursing, School of Nursing and Midwifery, Zabol University of Medical Sciences, Zabol 9861663335, Iran; morahnama0@gmail.com

**Keywords:** quality of care, doctor-patient, nurse-patient, holistic care, nephrology, experiences

## Abstract

This study aimed to describe the experiences of nurses in hemodialysis care. In this phenomenological study, purposive sampling began and continued until data saturation. The research environment was the Hemodialysis unit. Data was collected through semi-structured interviews. Finally, two main classes and four sub-classes were identified, including factors effective on care (inhibitors and facilitators) and care outcomes (the negative effects of care on the nurse and the positive effects of care on the patient), and “challenging care” as the main theme. As the results show, nurses suffer from several physical and mental harm, and this harm even extends to their family environment, and their families are indirectly affected by the negative effects of this care. Therefore, strengthening management approaches to eliminate the inhibitor factors is essential in order to prevent nurses’ burnout or quitting while improving the quality of care provided by them.

## 1. Introduction

Chronic kidney disease (CKD) includes a spectrum of various pathologic processes that can lead to irreversible reduction of renal function [1]. The prevalence of chronic kidney disease is increasing in the world [2]; the average global growth rate of this disease was 8% per year in the past five years. In Iran, this growth is higher than the global average and is about 12% [3]. Many patients with end-stage kidney disease (ESKD) cannot get kidney transplants and undergo hemodialysis for many years [4]. Since the first hemodialysis in humans by Hess in 1924, it is still considered the most important treatment for these patients [5]. In 2009, 92.9% of patients in the United States underwent hemodialysis [6]. In Iran, hemodialysis is the most common treatment for renal failure, and 50% of patients are treated with this method [7]. Although, hemodialysis reduces the disease symptoms and improves patients’ lifestyle, their quality of life is affected by the disease and its complications that lead to disability [8]. Meanwhile, hemodialysis imposes great stress on the patient, and patients undergoing it usually experience higher levels of psychological than physical stress [9]. Hemodialysis, therefore, requires specialized nursing care, including establishment of a therapeutic and interpersonal relationship, treatment of physical symptoms, and attention to the functional limitations, mental disorders, and educational needs of these patients [5]. Basically, nurses are the main people who provide care for these patients, and their most important responsibility is to identify the essential care of these patients [10]. Hemodialysis patients need mental support to adapt to their current status, and nurses can help them become accustomed to their problems and fears of the disease by reducing anxiety, enhancing adaptability, supporting decision making, and providing emotional support and education [11]. Therefore, nurses’ awareness of high quality of care can affect the care of these patients and increase patients’ satisfaction; notably, the quality of the provided nursing care is an important indicator of nurses’ involvement in the care program [9]. Shafipour reported in their qualitative study that patients receive comfort from humans more than environment and modern facilities [12]. This issue reflects the important role of nursing and human nursing in contrast to the need for technical nursing and modern specialized facilities [8]. Sometimes, however, nursing care lacks the necessary adequacy and safety of patients. Future studies should therefore aim to clarify the dimensions of nursing care plan and place the nursing care structure into standard care [13]. This, of course, requires identification of barriers to nursing care, as working in hemodialysis unit makes many nurses exhausted due to factors such as heavy workload and lack of resources [5]. Although nephrology nurses play an important role in determining the adequacy and quality of care in hemodialysis patients, few studies in Asia have examined the quality of nursing care. Meanwhile, adequacy of the provided care is assessed by quantitative measures rather than qualitative ones, while qualitative studies provide the best tool for understanding human experiences and is more appropriate for assessing the experiences and views of a group of people on a particular topic [9]. Qualitative research is based on the hypothesis that there is a dynamic truth and proposes a perspective for searching and understanding human elements that cannot be measured through quantitative research methods [14]. Therefore, considering the importance of the quality of nursing care and understanding deep experiences of nurses, as well as the literature review that indicated few studies on experiences of hemodialysis patients, this study aimed to describe the living experiences of nurses in hemodialysis care.

## 2. Experimental Section

This study was reported according to COREQ guideline [15]. This qualitative research used descriptive phenomenology with a Colaizzi approach. Descriptive phenomenology is a method for analyzing and describing specific phenomena free of any pre-judgment that shapes an understanding of living experiences at the time of attention and focuses on the richness, breadth, and depth of these experiences [16].

In this study, the research environment is the Hemodialysis Department of Amiralmomenin Hospital in Zabol, which was selected due to the cooperation of the relevant authorities and easier access to the research units. To select participants, purposive sampling began and continued until data saturation. In qualitative research, data saturation determines the sample size in phenomenology research [17]. The criterion for selecting nurses that participated in this study was having a bachelor’s degree and at least one year of work experience in hemodialysis department. The main data collection method in this study was semi-structured interviews. Open questions such as “Please describe the care you provide for hemodialysis patient during your work?” and “Define the memories you remember about this?” were used as interview guide question that were earlier tested on two persons as pilot. Interview is considered as the main method of data collection in phenomenological research and provides a situation for participants to describe their views on the world as they have experienced in their own language and vocabulary [17]. The interviewers (H.S. and M.R) had a lot of experience in qualitative research on spiritual care and conducted face to face interviews individually in one of the rooms of the hemodialysis units in a quiet environment. The duration of each interview was 45 min to 1 h in one or two sessions depending on the free time and the patience of the nurse, using audio recorder device. In total, until data saturation, nine participants were interviewed. The interviews were conducted in January 2017. All the selected nurses participated in interviews. The authors tried to maximize the diversity of participants (in terms of work history, age, gender, and marital status). All interviews were recorded and then handwritten immediately for analysis. Usually, those who work with descriptive phenomenological methods use Colaizzi technique for data analysis [16]. The seven-step Colaizzi technique involves studying participants’ descriptions in order to understand them, extracting the important sentences, formulating known meanings, categorizing the data, compiling results in the form of a comprehensive description, a clear statement of the basic structure of the studied phenomenon, and a final validation of the findings [14]. The same approach was used to analyze the data in this study. During this study, methods used to ensure the accuracy and robustness of data, including credibility, transferability, dependability, and confirmability, were considered as scientific accuracy criteria in qualitative research [14]. In order to confirm the findings’ acceptability, the researcher involved the research subject extensively, and the research findings were reviewed by the participants and other colleagues for approval. To confirm the transferability of the findings, the authors tried to use nurses with different demographic characteristics and different experiences. To confirm the coherence, the research findings were provided to another researcher who was not involved in this research, and his conclusions were compared with that of the study researcher. For confirmability, the findings were commonly evaluated by three faculty members and the researcher tried not to interfere with the assumptions in the process of data collection and analysis, as much as possible.

This study was approved by the University’s Ethics Committee (code: Zbmu.1.REC.1396.209). Before beginning the study, the consent of relevant authorities was obtained. At the beginning of the interview, the research objectives and the interview method were explained to the participants, and they were ensured about the confidentiality of their information and their choice to participate in the study. Then, informed consent was obtained from them. The interview time was adjusted based on the coordination and willingness of the participant so that it would not interfere with their daily schedules. MAXQDA 12 (VERBI Software Sozialforschung GmbH, Berlin, Germany) was used for data management.

## 3. Results

The demographic characteristics of the participants are presented in Table 1. Analysis of the data obtained from nurses regarding hemodialysis patient care led to the extraction of two main categories and four sub-class, including mutual factors effective on care (care inhibitors and facilitators) and care outcomes (negative effects of care on the nurse and positive effects of care on the patient), and “challenging care” was identified as the main theme (Table 2).

### 3.1. Mutual Factors Affecting Care

Nurses encounter two factors when caring for hemodialysis patients, which includes “care inhibitors” and “care facilitators”.

### 3.2. Care Inhibitors

Nurses’ experience in the hemodialysis department showed that they had many inhibitory factors in the process of providing care to patients. Some of them were related to nurses (shortage of nurses, nurses’ financial and family problems, inexperienced nurses, nurses’ fatigue and mental stresses, and heavy work shifts), while some factors were related to patients (patient’s emotional sensitivity and difficulty in attracting patients’ trust), and some factors were due to poor management (inadequate ventilation of the department, lack of equipment technician, shortage of devices and equipment, and weak cooperation of head nurse with nurses).

“If the number of devices and nurses increase proportional to the number of patients, it can greatly affect the quality of care” (A 38-year-old woman with 10 years of work experience).

“When I first came to hemodialysis department, early in my career, the patients did not accept me and did not trust me, so they did not let me do my job” (A 29-year-old woman with 2.5 years of work experience).

“The nurse’s experience has a positive effect on dialysis patients. For example, once an inexperienced nurse enters, she wants to detach the patient from the machine, and the patient needs resuscitation” (A 40-year-old woman with 18.5 years of work experience).

### 3.3. Care Facilitators

“Assessing nurses’ experience revealed that some factors can help in care of hemodialysis patients, including nurse’s experience, the emotional relationship between the nurse and patient, nurse’s high educational level, and a safe environment” (A 35-year-old woman with 10 years of work experience).

“I can gain my patient’s satisfaction by creating a safe and secure environment, and a close relationship with the patient” (A 38-years-old man with eight years of work experience).

“We need to raise our literacy level, provide training and retraining courses for care of dialysis patients, which has a great influence on perfect and high-quality care of these patients” (A 38-year-old man with eight years of work experience).

### 3.4. Mutual Care Outcomes

Nurses’ experience in hemodialysis department showed that there were pleasant and unpleasant outcomes, including “negative effects of care on the nurse” and “positive effects of care on the patient”.

### 3.5. The Negative Effects of Care on the Nurse

The experiences expressed by nurses suggest that taking care of hemodialysis patients is associated with negative personal effects (facing physical damages), mental effect (misconduct, bad temper), feelings of burnout, obsessive thoughts regarding health, feelings of depression and anxiety, tendency to leave the department, and negative family effects (neglecting children, inability to meet spouse’s needs, inability to perform housekeeping duties, and interference of professional with family responsibilities).

“We have a lot of work pressure in this department which makes us feel tired and angry; when I go home, I do not pay enough attention to my children, I cannot meet the needs of my spouse, and I have less patience in my housekeeping duties” (A 38-year-old woman with 10 years of work experience).

“Because we are dealing with patients’ blood in hemodialysis and take care of patients with hepatitis, we should be careful not to get needle stick” (A 38-year-old woman with 14 years of work experience).

“When I came to this department and saw the problems of these patients, I lost my mood and I feel I’m getting depressed” (A 27-year-old woman with four years of work experience).

### 3.6. Positive Effects of Care on the Patient

Examining nurses’ experiences showed the positive effects of care provided by nurses on patients including routine care (care before, during and after dialysis; education; referral), reduction of physical problems, reduction of complications, improvement of the patient’s mental state and sense of safety, increase in the patient’s life expectancy, and interdependencies between the patient and nurse.

“When we take good care of patients, it affects their general condition and makes them feel safer” (A 29-year-old woman with 2.5 years of work experience).

“When you take care of patients well, it has a great impact on their physical condition and reduction of complications, and even the patients’ mood” (A 27-year-old woman with four years of work experience).

“Our nursing care offers joy and life expectancy to patients” (A 32-year-old woman with four years of work experience).

## 4. Discussion

Nurses’ experience in hemodialysis department showed that this care was influenced by mutual factors, some of which were “inhibitors” and some “facilitators”, and the care outcomes included “positive effects of care on the patient” and “negative effects of care on the nurse”. This section discusses the research findings.

In the present study, several factors were identified as care inhibitors; some were related to nurses and patients, and some were related to management failure. Several studies have suggested these inhibitory factors. Namnabati pointed to inadequate nursing skills as one of the care challenges [18], Ebadi referred to the shortage of nurses in Iran as a serious challenge [19], and Novoboar pointed at the end of their study that the shortage of nurses and lack of staffing, called nurse assistance, are inhibitory factors in the care of hemodialysis patients [20]. Masoumi referred to heavy workloads, various job shifts, and lack of mental support as nurses’ stressors, which reduced the quality of patients’ care [21]. Ndambuki also emphasized that hospitals should increase the number of nurses and their devices in order to maintain the level of satisfaction in renal patients [22]. Dehghan Naiery mentioned in their research that providing adequate nursing staff and equipment are important factors in the prevention of missed nursing care that refers to undone and delayed care [23].

It was shown that high levels of nurse’s experience, the emotional relationship between the nurse and patient, high educational level of the nurse, and safe environment can facilitate care. Several studies have identified these facilitators. Confirming the importance of the emotional relationship between the nurse and patient, Zamanzadeh referred to compassionate care as the constructive interaction between the nurse and patient, during which the nurse places himself in the patient’s shoes and understands his circumstances, to discover his concerns [24]. Moreover, Atashzadeh and colleagues identified meeting the patients’ needs through communication, support, and mutual respect between the nurse and the patient as purposeful care [25], and Baljani declared, as a result of their study, that in order to meet the patients’ needs, it is necessary to emphasize the emotional and social aspects of care in nursing education and planning [26]. Moran, in their study on the need for increasing nurses’ awareness, pointed out the importance of effective communication in providing supportive care for renal patients [27]. Confirming the importance of the nurse’s work experience, Mohammadi declared that more experienced people in the workplace can have a stronger supportive role than other people in therapeutic team, especially those with low experience [28], and regarding the need for a safe healthcare environment, Nobahar in their qualitative study aimed at explaining the experiences of patients, nurses, caregivers, and doctors in hemodialysis department in the quality of nursing care mentioned environment as one of the important factors in this regard [9]. Mahdavi Shahri also suggested that the quality of care can improve through environmental monitoring and creating a pleasant environment with the least stress possible [29].

Confirming the importance of educational level of the nurse, Nobahar named nurses’ basic knowledge as a facilitator of hemodialysis patient care [20]. As a result of their study, Berzou also identified nurses’ knowledge as an effective factor in providing patient’s comfort during hemodialysis [30].

In the present study, the nurses stated that providing care in hemodialysis department has negative personal effects, including physical and mental harm. Confirming this conclusion, Naidoo stated that intensive care unit nurses need to provide care and give physical and psychological support [31]. Depression was another experience of the nurses in the present study. Kazemi Golghahi also suggested that the prevalence of depression was significantly higher in the studied nurses than the general population. They concluded that as far as nurses are at high levels of stress and severe workload, planning for reducing the prevalence of depression plays an important role in patients’ health [32]. Also, Letvak reported that the prevalence of depression was higher than normal in the studied nurses [33]. Feeling exhaustion was another experience of nurses in the present study. Covermasi reported a moderate level of burnout among nurses in the hemodialysis department in their study [34]. Negative family effects included negative job effects of the studied nurses. Tavangar also confirmed these negative job effects in their study. Nurses, while being familiar with various forms of work/family conflicts, should learn methods to confront them in order to minimize their negative consequences [35].

The experience of nurses showed that the outcome of the care provided by them was positive for patients. Confirming this conclusion, Castner reported that nephrology nurses can be effective in reducing patients’ problems by performing interventions before, during and after dialysis [36]. Khoieniha also emphasized that nurses are the largest occupational healthcare group with a significant potential for influencing the quality of healthcare services [37]. Asgari suggested in their qualitative research that, from the perspective of hemodialysis patients, appropriate nurses’ supportive behavior plays an important role in feelings of relaxing and comfort, safety and confidence, and speeds up adaptation to hemodialysis [38]. The interdependence between the patient and the nurse was one of the findings of this study, but it is contrast to the results of Moran on hemodialysis patients who were not satisfied with nurses’ communication and stated that nurses rarely communicated with them and mainly paid attention to the physical and technical aspects of care [27]. The differences in the research environments and cultural differences may play a role in this difference.

## 5. Conclusions

The experience of nurses that participated in this study provided a clear picture of the care provided in the hemodialysis department of Amir al-Momenin Hospital in Zabol and clarified that the care provided by these nurses was a challenging one, which is influenced by mutual factors, some of which are inhibitors and some facilitators. The mutual influence of these factors prevents the care outcome from being completely positive. In addition to reducing the physical and psychological problems of patients and increasing their sense of safety and hope, which leads to a sense of dependency between patients and nurses, the caring nurses suffered many physical and mental harms, and this damage even extends to their family environment: their family members were also indirectly affected the negative effects of this care. Therefore, strengthening managerial approaches toward eliminating the inhibitory factors by addressing deficiencies (shortage of manpower, eliminating department’s shortages, and providing them with new equipment) can help resolve nurses’ problems, and increasing nurses’ knowledge and experience in communication with hemodialysis patients seems necessary to resolve the challenges in the care of hemodialysis patients in order to improve the quality of care provided by nurses and prevent burnout.

## Figures and Tables

**Table 1:** Demographic characteristics of participants (nurses in hemodialysis department).

Demographics		Number (Percentage)/Mean (SD)
Sex	Female	7 patients (77.8%)
	Male	2 patients (22.2%)
Marital status	Single	2 patients (22.2%)
	Married	7 patients (77.8%)
Age		(28–53 years) 33.7 (5.4)
Experience in hemodialysis department		4.7 years (3.7)

**Table 2:** Main theme, main classes, and subclasses derived from data.

Main Theme	Main Class	Subclass	Codes
Challenging care	Mutual factors affecting care	Care inhibitors	Related to nursing (shortage of nurse, financial problems of nurses, family problems, inexperienced nurses, nurse’s fatigue and mental pressure, and heavy work shifts)
			Related to patients (hard to gain the trust of patients and emotional sensitivity of patients)
			Due to poor management (poor ventilation, lack of equipment technicians, shortage of equipment and devices, poor cooperation of head nurses with nurses)
		Care facilitators	Nurse’s high experience, emotional relationship between the nurse and the patient, high educational level of the nurse, and safe therapeutic environment
	Care outcomes	Negative effects of care on the nurse	Negative personal physical effects (facing physical harms) and emotional (irritability, bad temper), exhaustion, obsessive thoughts regarding health, feeling of depression)
			Negative family effects (neglecting children, failure to meet spouse’s needs, and inability to handle housekeeping duties)
		Positive effects of care on the patient	Reduced physical problems, reduced complications, improving the patient’s mental state and feeling safe, increasing patient’s life expectancy and interdependencies between the patient and the nurse
